# Feasibility of chemosensitivity testing in soft tissue sarcomas

**DOI:** 10.1186/1477-7819-3-20

**Published:** 2005-04-18

**Authors:** Marcus Lehnhardt, Thomas Muehlberger, Cornelius Kuhnen, Daniel Brett, Hans U Steinau, Hamid Joneidi Jafari, Lars Steinstraesser, Oliver Müller, Heinz H Homann

**Affiliations:** 1 Department of Plastic Surgery, Burn Center, Hand surgery, Sarcoma Reference Center, BG University Hospital Bergmannsheil, Ruhr University Bochum, Bürkle-de-la Camp Platz 1, 44789 Bochum, Germany; 2 Institute of Pathology, BG University Hospital Bergmannsheil, Ruhr University Bochum, Germany; 3 Tumor Genetics Group, Max-Planck-Institut für molekulare Physiologie, Dortmund, Germany

**Keywords:** soft tissue sarcoma, chemotherapy, chemosensitivity, ATP-TCA

## Abstract

**Background:**

Soft tissue sarcomas comprise less than 1% of all solid malignancies. The presentation and behavior of these tumors differs depending on location and histological characteristics. Standard therapy consists of complete surgical resection in combination with adjuvant radiotherapy. The role of chemotherapy is not clearly defined and is largely restricted to clinical trials. Only a limited number of agents have proved to be effective in soft tissue sarcomas. The use of doxorubicin, epirubicin and ifosfamide allowed response rates of more than 20%. In addition, recent chemotherapy trials did not demonstrate any significant differences in efficacy for various histological subtypes.

**Methods:**

The objective of this study was to gain additional information about the chemosensitivity of soft tissue sarcomas to seven 7 different chemotherapy agents as single drugs and 4 combinations. Therefore we used an established ATP based in-vitro testing system and examined 50 soft tissue sarcomas. Chemosensitivity was assessed using a luciferin-luciferase-based luminescence assay providing individual chemosensitivity indices for each agent tested.

**Results:**

The sensitivity varied widely according to the histological subtypes. The tumors state of cellular dedifferentiation played a crucial role for the efficiency of the chemotherapeutic agents. The sensitivity also depended on the presentation of the sarcoma as a primary or recurrent tumor. The highest sensitivity was demonstrated for actinomycin D as a single agent, with 74% of the tumor samples exhibiting a high-grade sensitivity (20% low sensitivity, no resistance). The combination of actinomycin D and ifosfamide yielded a high sensitivity in 76% (2% resistance). Doxorubicin as a mono-therapy or in combination with ifosfamide achieved high sensitivity in 70% and 72%, respectively, and resistance in 6% of the samples.

**Conclusion:**

Chemosensitivity testing is feasible in soft tissue sarcomas. It can be used to create sensitivity and resistance profiles of established and new cytotoxic agents and their combinations in soft tissue sarcomas. Our data demonstrate measurable discrepancies of the drug efficiency in soft tissue sarcomas, sarcoma subtypes and tumor recurrencies. However, current therapeutic regime does not take this in consideration, yet.

## Background

Soft tissue sarcomas account for less than 1% of malignant neoplasms. Approximately 140 different histological types of sarcomas have been described [1]. Sarcomas arise in tissues of mesenchymal or ectodermal origin and may thus occur anywhere in the body The surgical treatment of choice is the wide surgical excision of the tumor in combination with radiotherapy [2,3]. Currently, about 30–50% of all patients die within 5 years of primary diagnosis. This overall poor prognosis is mainly due to metastatic disease at the time of diagnosis. Approximately 40% of patients with high-grade sarcomas develop pulmonary metastases in spite of local tumor control [4]. The median duration of survival with apparent metastases is presently 8–12 months [2,4].

Soft tissue sarcomas are notoriously resistant against most chemotherapeutic agents [5]. As first line treatment, only doxorubicin (adriamycin), epirubicin and ifosfamide have achieved a single-agent activity of more than 15% resulting in response rates of 18%-29% [6,7]. The combination of anthracyclines and ifosfamide resulted in response rates of 40%-50%, with 10% of patients in complete remission [8]. ite the reported association between remission and prolonged survival, an overall superiority of combination chemotherapy over the administration of single-agent doxorubicin has not been established yet. Ifosfamide can play a major role as a second-line treatment with response rates of up to 30%-50% following the failure of anthracylin [2,7].

Despite the disparate appearance and histology of sarcomas, only few drugs and combinations have been used as treatment regimes [5,9].

Recently, new strategies have been pursued to improve treatment options for well defined subgroups of sarcomas. One example is the successful use of imatinib hydrolasis, a specific tyrosine kinase inhibitor, in gastrointestinal stromal tumors [10,11].

Several new drugs such as exatecan, TZT 1027, trofosfamid and topotecan are under investigation [12-15]. Another promising agent for the treatment of soft tissue sarcomas is ET-743. Several clinical trials have shown response rates of up to 20% in untreated and 10% in pretreated sarcoma patients. Approximately 50% of patients in these series have shown long-lasting stabilization of disease [4,9,16,17].

**Figure 1:**
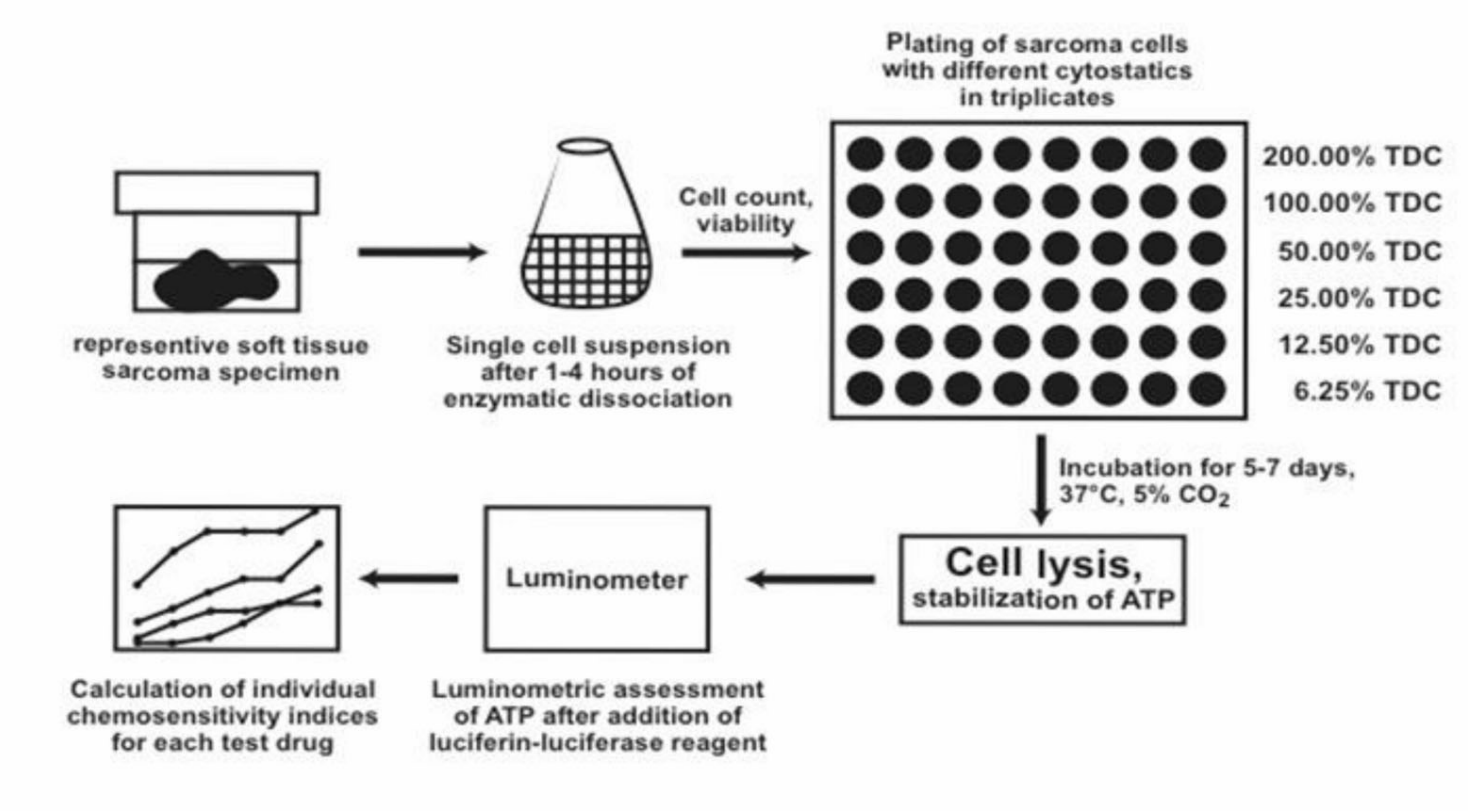
Principle of chemosensitivity testing using the ATP luminescence method (ATP-TCA). TDC, test drug concentration

In the present study we used a testing system, the ATP-based tumor sensitivity assay (ATP-TCA) (Fig. 1) The successful use of ATP-TCA has been demonstrated for pretesting of chemosensitivity in melanoma, breast and ovarian cancer [18-20] as well as other tumors [21-26]. Based on these results, we performed in vitro chemosensitivity testing with the ATP-TCA in patients with soft tissue sarcoma. The aim of this study was to evaluate the feasibility of ATP-TCA in soft tissue sarcomas and to gain information about their chemosensitivity profiles with regard to subtype, grade of differentiation and tumor recurrence.

### Patients and Methods

After informed consent was obtained, the tumors were resected in 50 patients with diagnosed sarcomas of the extremities. The group of tumors included liposarcomas (n = 17), malignant fibrous histiocytomas (MFH/NOS; n = 16), extraskeletal chondrosarcomas (n = 8), rhabdomyosarcomas (n = 5) and malignant peripheral nerve sheet tumors (MPNST;n = 4). Clinical staging of the patients was performed in accordance with the criteria of the American Joint Committee on Cancer. The mean age of the patients was 63 years, ranging from 32–76 years. The sex distribution was 20 females and 30 males. The sarcomas were primary tumors in 21 patients and recurrencies in 29 patients. None of the patients had received adjuvant chemotherapy, neither before nor after surgery. All patients with recurrent tumors had radiotherapy following the excision of the tumor. All patients underwent wide, complete resection of the tumor. Histological grading showed GII in 17 tumors and GIII in 33 tumors (Tab. 1).

**Table 1:** Basic data of patients with soft tissue sarcomas (n = 50)

	n	age (mean-range)	primary tumors	recurrent tumors	grade G2	grade G3
Liposarcoma	17	61 (32–73)	9	8	6	11
NOS-Sarcoma/MFH	16	64 (42–69)	7	9	7	9
Extraskeletal Chondrosarcoma	8	65 (62–72)	1	7	2	6
Rhabdomyosarcoma	5	68 (64–76)	4	1	1	4
MPNST	4	59 (55–67)	0	4	1	3

Tumor tissue was sampled under sterile conditions by a histopathologist and stored in cell culture medium (DMEM, Sigma™, Germany) with antibiotics (100 U/ml penicillin and 100 μg/ml streptomycin) at 4°C. In vitro chemosensitivity testing was performed with a ATP-based assay (ATP-TCA, DCS Innovative Diagnostic Systems^®^, Hamburg, Germany) [27]. At least 1 cm^3 ^of tumor tissue was minced and dissociated enzymatically. The single-cell suspension was depleted of red blood cells and cellular debris through Ficoll-Hypaque density gradient centrifugation. After assessing the viability of the cells, a cell count was conducted. The cells were then incubated in polypropylene round-bottom 96-well plates containing 2 × 10^4 ^cells per well. Different cytotoxic agents in different concentrations were added to the wells, whereas some wells received no supplements and served as negative controls. Applied chemotherapeutics were doxorubicin, epirubicin, ifosfamide, dacarbazine (DTIC), actinomycin D, cisplatin and vincristine at six different dilutions (6.25–200%) of the test drug concentrations (TDC) shown in Tab. 2.

**Table 2:** Cytotoxic drugs used in the ATP-TCA

drug	100% TDC* (μg/ml)
doxorubicin (adriamycin)	0,5
epirubicin	0,5
ifosfamide (mafosfamide)	3,0
dacarbazine (DTIC)	10,0
actinomycin D	0,1
cisplatin	3
vincristine	0,4

*TDC, test drug concentration

While the concentration of a chemotherapeutic agent is typically measured in plasma or serum with defined precision, sensitivity, and specificity, it is possible to clinically and analytically validate the data to be used as test drug concentration in other biological samples or in cell cultures . The individual TDC for each cytotoxic drug was determined previously (Andreotti et al. 1995) by reference to known pharmacokinetic and response data. After 7 days of incubation at 37°C, 5% CO_2 _and 100% humidity, the cells were lysed and the ATP content of each well was measured by a luciferin-luciferase-based luminescence assay with a Dynatech ML1000 luminometer (Fig. 1). Individual sensitivity indices for each test drug or drug combinations were calculated from the obtained data curves by summing up the percentages of tumor inhibition for each drug concentration tested (6.25%, 12.5%, 25%, 50%, 100%, 200%) followed by the subtraction of 600. A sensitivity index of 600 indicates unrestrained tumor cell growth and minimal drug sensitivity, whereas a sensitivity index of 0 reflects a complete tumor inhibition and maximal drug sensitivity. Moreover, the chemosensitivity of the test samples was classified according to the tumor inhibition at different TDC. The chemotherapeutic single agents and combinations were arranged in groups starting from high sensitivity, over moderate sensitivity and low sensitivity, to resistant. The grouping was performed according to the threshold values shown in Tab. 3.

**Table 3:** Classification of ATP-TCA chemosensitivity testing results

	Tumor growth inhibition at 200% TDC*	Tumor growth inhibition at 25% TDC*
High sensitivity	>95%	>70%
Moderate sensitivity	>95%	50–70%
Low sensitivity	>95%	<50%
	<95%	>50%
Resistant	<95%	<50%

*TDC, test drug concentration

### Statistical analysis

The statistical significance of differences between sub entities of soft tissue sarcomas, tumor grading and the comparison between primary and recurrent tumors was calculated by the Mann-Whitney-U-test and the Chi squared test. *P *< 0.05 was considered statistically significant.

## Results

ATP-TCA testing was done on 53 tumor samples from 53 patients. 50 (94%) of these tumor samples were included in the study. 3 samples had to be excluded due to bacterial infection (n = 2) or mycosis (n = 1). The sensitivity profiles of the sarcomas exhibited a significant heterogeneity in response to the array of different cytotoxic agents (Tab. 4)

**Table 4:** Chemosensitivity testing in 50 soft tissue sarcoma patients

Cytotoxics	Resistant	Low Sensitivity	Moderate Sensitivity	High Sensitivity	Mean Sensitivity Index*
Actinomycin D	00/50 (00%)	10/50 (20%)	03/50 (06%)	37/50 (74%)	137
Doxorubicin (Adriamycin)	03/50 (06%)	02/50 (04%)	10/50 (20%)	35/50 (70%)	140
Ifosfamide	10/50 (32%)	00/50 (00%)	02/50 (04%)	32/50 (64%)	234
Epirubicin	08/50 (16%)	00/50 (00%)	12/50 (24%)	30/50 (60%)	245
Cisplatin	39/50 (82%)	11/50 (22%)	00/50 (00%)	00/50 (00%)	444
Dacarbazine	45/50 (95%)	05/50 (5%)	00/50 (00%)	00/50 (00%)	532
Vincristine	42/50 (84%)	08/50 (16%)	00/50 (00%)	00/50 (00%)	513
Doxorucicin + Ifosfamide	03/50 (06%)	00/50 (00%)	11/50 (22%)	36/50 (72%)	139
Actinomycin D + Ifosfamide	01/50 (2%)	08/50 (16%)	03/50 (06%)	38/50 (76%)	163
VAC**	03/50 (06%)	05/50 (10%)	07/50 (14%)	35/50 (70%)	218
CYVADIC***	02/50 (04%)	00/50 (00%)	15/50 (30%)	33/50 (66%)	255

*Data are means from individual chemosensitivity indices for each test drug or drug combination, which were calculated by summing up the tumor growth inhibition for each drug concentration tested followed by the subtraction of 600. A sensitivity index of 600 indicates unrestrained tumor cell growth and minimal drug sensitivity, whereas a sensitivity index of 0 reflects complete tumor growth inhibition and maximal drug sensitivity.

**VAC: Vincristine, Actinomycin D, Cyclophosphamide

***CYVADIC: Cyclophosphamide, Vincristine, Doxorubicin, Dacarbazine

**Figure 2:**
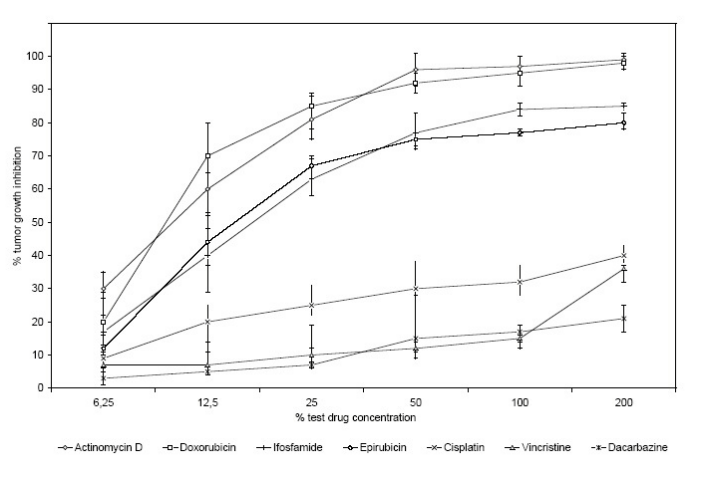
Original data curves obtained with ATP-TCA in 50 soft tissue sarcomas. Each plot shows the corresponding test results with seven different cytotoxics as single agents.

The strongest chemoresistance was demonstrated for the single use of dacarbazine with 95% of the samples being resistant. The application of vincristine and cisplatin resulted in rates of resistance of 84% and 82%, respectively. The highest response rate was found for actinomycin D, with 74% of the samples showing a high sensitivity without any resistances. These positive results were followed by doxorubicin and ifosfamide with a highly sensitive response in 70% and 64% and rates of resistance in 6% and 12%, respectively. In contrast, epirubicin had a markedly lower effect on inhibition, with high sensitivity in 60% and resistance in 16% of cases (Tab. 4). The tumor inhibition of the corresponding concentration of the 7 single agent chemotherapeutics is shown in Fig. 2. It reveals the superior efficiency of actinomycin D, doxorubicin, dacarbazin and ifosfamide, compared to cisplatin, vincristine and dacarbazin (Fig. 2).

**Figure 4:**
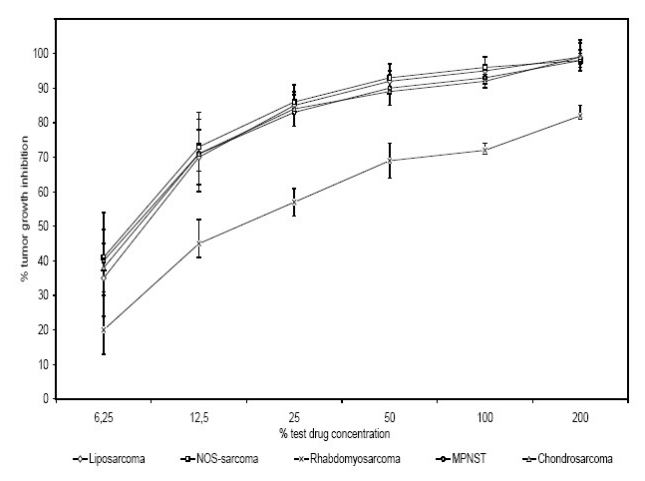
Effect of Actinomycin D as single agent measured by ATP-TCA in different types of soft tissue sarcomas (Liposarcomas:n = 17, NOS-Sarcomas/MFH:n = 16, Chondrosarcomas:n = 8, Rhabdomyosarcomas:n = 5, MPNST:n = 4) (p < 0.05).

In addition to single drug testing, four different combinations of agents were submitted to ATP-TCA testing. Chemosensitivity was less pronounced for CYVADIC (cyclophosphamide, vincristine, doxorubicin and dacabazine) with 66% of the samples showing high sensitivity versus 4% of resistant samples. The VAC combination (vincristine, actinomycin D and cyclophosphamide) resulted in 70% of high sensitivity and 6% resistance. Doxorubicin and ifosfamide in combination yielded highly sensitive responses in 72% and 6% of resistant cases. The combined use of actinomycin D and ifosfamide resulted in the overall strongest chemosensitivity of all combinations used with 76% of high sensitivity and 2% of resistance (Tab. 4). The tumor inhibition of the tested chemotherapeutic agent combinations is demonstrated in correlation to the agent's concentrations in Fig. 4.

The cytotoxic treatment with actinomycin D resulted in significantly divergent profiles of chemosensitivity depending on the histological type of soft tissue sarcoma (Fig. 4). Rhabdomyosarcomas were especially chemoresistant against actinomycin D (p < 0.05).

This is in contrast to the response pattern of other types of sarcomas to this particular chemotherapy. There was no difference in chemosensitivity between rhabdomyosarcomas and other tumor types for the remaining cytotoxic drugs used.

**Figure 5:**
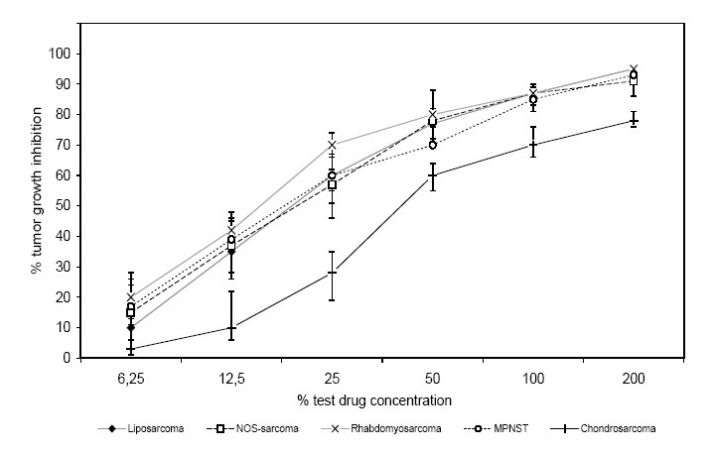
Effect of Doxorubicin (Adriamycin) as single agent measured by ATP-TCA in different types of soft tissue sarcomas (Liposarcomas:n = 17, NOS-Sarcomas/MFH:n = 16, Chondrosarcomas:n = 8, Rhabdomyosarcomas:n = 5, MPNST:n = 4) (p < 0.05).

Complementary, doxorubicin revealed significantly lower cytotoxic effect on extraskeletal chondrosarcomas compared to all the other tumors (p < 0.05) (Fig. 5). The remaining chemotherapeutic agents showed no difference in chemosensitivity between extraskeletal chondrosarcomas and the other tumor sub entities tested.

**Figure 6:**
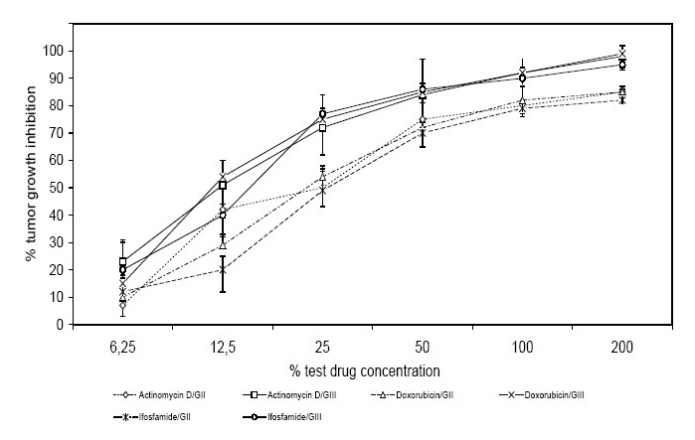
Chemosensitivity profiles of 50 soft tissue sarcomas divided by tumor grade: G2 (n = 17), G3 (n = 33). Results shown for Actinomycin D, Doxorubicin and Ifosfamide (p < 0.05).

There also was a strong statistical correlation between the histological grading and the chemosensitivity. GIII tumors (n = 33) demonstrated a significantly higher chemosensitivity for actinomycin D, doxorubicin and ifosfamide than GII tumors did (p < 0.05) (n = 17) (Fig. 6).

**Figure 7:**
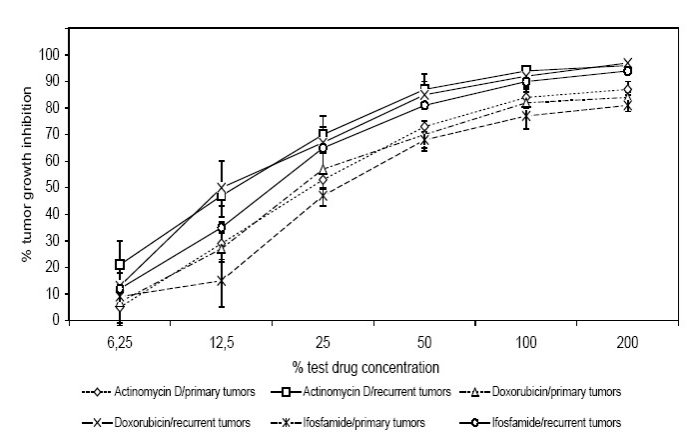
Chemosensitivity profiles of 50 soft tissue sarcomas divided by primary- (n = 21) and recurrent- (n = 29) tumors. Results shown for Actinomycin D, Doxorubicin and Ifosfamide (p < 0.05).

Similar results were seen when chemosensitivity of primary tumors was compared to sensitivity of recurrent tumors. We found the recurrent tumors to be more chemosensitive to actinomycin D, doxorubicin and ifosfamide than the primary ones (p < 0.05) (Fig. 7).

## Discussion

In view of the limited number of effective chemotherapeutic drugs with response rates of less than 20%, the need for more detailed information about the tumor specific chemoresistance seems obvious [4,5,28]. A testing system would have to produce repeatable and comparable results of the chemosensitivity of various tumor types. Chemosensitivity testing with ATP-TCA proved a success in acquiring resistance profiles for different chemotherapeutic agents in metastasizing carcinoma of the breast [21] and recurrent tumors of the ovaries [24]. In these tumors, the intra- and inter-assay variability was less than 15%, which demonstrated a high sensitivity and linearity [29]. In the present study, the ATP-TCA test was practicable in 94% of all cases and thus represented a feasible method in soft tissue sarcomas.

Only a limited number of cytotoxic drugs, such as doxorubicin, epirubicin and ifosfamide, have been reported to produce response rates of more than 15% in the treatment of sarcomas, either as single agents or as part of drug combinations. There are no clinical trials providing evidence of prolonged survival rates after either adjuvant or neoadjuvant chemotherapy [30-32]. Currently, the EORTC is not conducting any clinical trials on the efficacy of chemotherapy in soft tissue sarcomas due to the lack of a prospected benefit. Chemotherapy is presently used in metastasized disease only which may account for the low acceptance of this therapeutic option in adult soft tissue sarcoma [30,33-36].

Further limiting factors regarding the feasibility of large-scale trials are the low incidence of sarcomas and the enormous heterogeneity of the types of tumors. At the moment, pathologists know more than 140 entities that may explain some problems of classification. The application of chemotherapy is thus not differentiated according to the histological type of tumor, yet.

Doxorubicin has been reported to achieve response rates of 20% to 26% and represents the most effective chemotherapeutic agent among the available first line single drug therapies [37]. In our study, the ATP-TCA testing of doxorubicin showed that 70% of tumors reacted highly sensitive. However, among all cytotoxic agents tested, doxorubicin proved to be only a second rate treatment regimen. The overall highest sensitivity of all single therapeutic agents was demonstrated for actinomycin D. This is in contrast to the poor clinical response rates of approximately 17% rendered by this drug [15,38]. Especially actinomycin D is in common use as single agent and combination chemotherapy for treatment of rhabdomyosarcomas in childhood and hyperthermia/isolated limb perfusion [15,39-41].

Ifosfamide serves as a prime drug in first line treatments under clinical conditions and as a high-dose cytotoxic agent in second line applications with response rates of more than 25% [7,42-45]. Yet, the ATP-TCA chemosensitivity testing found it to be significantly inferior to actinomycin D and doxorubicin.

The ATP-TCA confirmed strong chemoresistance of soft tissue sarcomas, known from several clinical trials for vincristine, cisplatin and dacarbazine as single agents [2,32,42].

**Figure 3:**
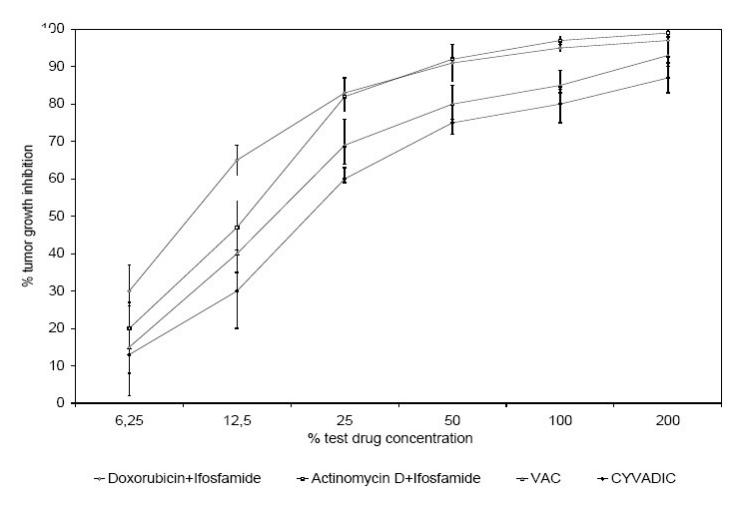
Original data curves obtained with ATP-TCA in 50 soft tissue sarcomas. Each plot shows the corresponding test results with four different drug combinations.

Similar results were found for combined chemotherapies. The CYVADIC combination of Gottlieb has been in use for over a decade [46], however, the ATP-TCA testing showed significantly higher chemosensitivities with the combination of actinomycin D and ifosfamide (Fig. 3).

The analysis of respective histological entities showed, that specific types of tumors respond in different ways. Rhabdomyosarcomas were significantly more resistant against actinomycin D than any of the other tumors tested (Fig. 4). Another example of sub-entity-dependant resistance was found in the chemosensitivity of extraskeletal chondrosarcomas on doxorubicin. The ATP-TCA testing showed a considerable chemoresistance of these tumors against doxorubicin, when compared to other types of soft tissue sarcomas (Fig. 5).

Different test results were also found depending on grading and whether the sarcoma presented as a primary or a recurrent tumor.

The results outlined in Fig. 7 show that the chemoresistance of primary tumors was considerably higher than the resistance of recurrent tumors. These results correspond to the regularly higher differentiation and the lower turn over time of primary tumors compared to recurrent tumors. The superior chemosensitivity of recurrent tumors is in sharp contrast to the worse prognosis of recurrencies [47-51].

GII tumors (n = 17) exhibited a significantly higher chemoresistance when compared to GIII tumors (n = 33) (Fig. 6). This could be due to the higher mitotic activity of tumor cells in the GIII group. The test results stress the importance of the state of cellular differentiation as a crucial parameter for the responsiveness of soft tissue sarcomas to cytotoxic therapies [32,36].

The results indicate that protocols for chemotherapy should be designed based on tumor specificity, sub entity specificity and according to the tumors grading to improve the response rates of soft tissue sarcomas.

## Conclusion

The pre-therapeutic chemosensitivity testing of cytotoxics with ATP-TCA offers the opportunity of improving the hitherto moderate success rates of conventional chemotherapy in soft tissue sarcomas. Sarcomas usually are of sufficient size to gain enough tissue for the testing procedure, a problem often encountered with small carcinomas of the breast. The test poses no risk or disadvantage for the patient and should be performed in cooperation with the oncologist.

To create an individualized chemotherapy protocol, evaluation of ATP-TCA chemosensitivity testing correlated with clinical data and in-vitro results are required. Due to the limited course of time of this study, the results were not yet associated with data of clinical responses in long-term follow-up observations. Future trials on any positive correlations of this kind should prove the validity of the testing method.

## Authors' contributions

ML: coordinated the work, developed the study design and prepared the manuscript

TM: prepared the manuscript and figures carried out statistical analyses,

CK: carried out histopathology of the tumors

DB: carried out ATP-TCA-Assays

HUS: developed the idea, have given final approval of the version to be published

HJ: carried out ATP-TCA-Assays

LS: carried out ATP-TCA-Assays

OM: conceived the study, have given substantial contribution to conception and design

HHH: conceived the work and participated in study design, gave final approval

## Competing interests

The author(s) declare that they have no competing interests.
